# Tourettic OCD: Current understanding and treatment challenges of a unique endophenotype

**DOI:** 10.3389/fpsyt.2022.929526

**Published:** 2022-07-27

**Authors:** Tamar C. Katz, Thanh Hoa Bui, Jennifer Worhach, Gabrielle Bogut, Kinga K. Tomczak

**Affiliations:** ^1^Department of Psychiatry, Boston Children’s Hospital, Boston, MA, United States; ^2^Tic Disorders and Tourette Syndrome Program, Department of Neurology, Boston Children’s Hospital, Boston, MA, United States

**Keywords:** Tourette syndrome, obsessive-compulsive disorder, OCD, TOCD, Tourettic OCD

## Abstract

Obsessive compulsive disorder (OCD) and chronic tic disorders (CTD) including Tourette Syndrome (TS) are often comorbid conditions. While some patients present with distinct symptoms of CTD and/or OCD, a subset of patients demonstrate a unique overlap of symptoms, known as Tourettic OCD (TOCD), in which tics, compulsions, and their preceding premonitory urges are overlapping and tightly intertwined. The specific behaviors seen in TOCD are typically complex tic-like behaviors although with a compulsive and partially anxious nature reminiscent of OCD. TOCD is not classified within the Diagnostic and Statistical Manual of Mental Disorders fifth edition (DSM-5) as an independent diagnostic entity, but mounting evidence suggests that TOCD is an intermediate neuropsychiatric disorder distinct from either TS or OCD alone and as such represents a unique phenomenology. In this review of TOCD we discuss clinical, genetic, environmental, neurodevelopmental, and neurocircuit-based research to better characterize our current understanding of this disorder. TOCD is characterized by earlier age of onset, male predominance, and specific symptom clusters such as lower tendency toward compulsions related to checking, cleaning, and reassurance seeking and higher tendency toward compulsions such as rubbing, tapping, or touching associated with symmetry concerns or thoughts of exactness. Functional magnetic resonance imaging (fMRI) imaging suggests that TOCD symptoms may arise from involvement of an intermediate neurocircuitry distinct from classic OCD or classic CTD. Small cumulative contributions from multiple genetic loci have been implicated, as have environmental factors such as infection and perinatal trauma. In addition, this review addresses the treatment of TOCD which is especially complex and often treatment resistant and requires pharmacology and behavioral therapy in multiple modalities. Given the distressing impact of TOCD on patients’ functioning, the goal of this review is to raise awareness of this distinct entity toward the goal of improving standards of care.

## Introduction

Obsessive compulsive disorder (OCD) and tic disorders such as Tourette Syndrome (TS) are well established entities within the pediatric population that can occur independently, but are often comorbid within the same individual. Over the last 10–15 years, there has been increasing awareness of the overlapping neurocircuitry of tics and OCD and the existence of an intermediate phenotype, known as Tourettic OCD (TOCD), in which symptoms are influenced by features of both OCD and TS and differ from either disorder alone (1). Patients with TOCD present with thoughts, sensations, and behavioral urges at the interface of compulsions and tics that may pose challenges to assessment, diagnosis, and treatment. Given that treatment differs for TS and OCD, combined therapy is typically required for full remission of TOCD symptoms.

Unlike the DSM-4, which classified OCD within the spectrum of anxiety disorders, DSM-5 delineates a distinct diagnostic classification of Obsessive-Compulsive and Related Disorders which encompasses OCD as well as body dysmorphic disorder, trichotillomania, hoarding disorder, and excoriation disorders (2). Despite this new classification, TOCD is not recognized as an independent diagnostic entity within DSM-5. Instead, a qualifying specification of “Tic-related” OCD is suggested, although this qualifier simply indicates any current or past history of a tic disorder but does not illuminate the unique nature of TOCD symptoms. This distinction is important as the symptoms of TOCD do not equate to simply having a comorbid tic disorder and OCD.

The goal of this review is to expand upon the original framework of TOCD (1) as an independent diagnosis with updated clinical, genetic, neurodevelopmental, and neurocircuit-based research as they have evolved over the past 15 years since the original conceptualization of TOCD. As tics are typically childhood onset disorders, tic-related OCD and TOCD are predominantly pediatric diagnoses and may represent developmentally unique subtypes of pediatric OCD. The overlapping neurocircuitry of tics and OCD during key developmental stages argues toward an intermediary neuropsychiatric disorder that may later resolve as brain architecture matures and tics are often outgrown, leading to changes in the nature of OCD symptoms as patients age.

## Symptom presentation

To understand the unique nature of TOCD symptoms it is helpful to first elucidate the symptoms of TS or OCD alone.

Tics are sudden, stereotyped movements, typically repetitive, that wax and wane in severity and intensity. Typically involuntary, they are at times associated with a preceding somatic sensation, often described as a physical or sensory urge that is relieved by engaging in either a motor movement or phonic vocalization. Examples of classic tics may include but are not limited to: facial grimacing, shoulder shrugging, tapping, touching, blinking, neck jerking, or vocalizations such as throat clearing, coughing, whistling, grunting, or yelling words out of context.

In contrast, OCD is categorized by the presence of intrusive, ego-dystonic thoughts, known as *obsessions* that are distressing to the individual and cause heightened anxiety. These are commonly followed by *compulsions*, which are repetitive ritualized actions intended to alleviate the anxiety. A classic example would be contamination fears that lead to compulsive washing behaviors. Obsessions typically recur throughout the day and lead to compulsions which can last for hours and interfere with functioning in multiple domains including academic, social, emotional, or physical. In more severe cases, compulsions may interfere with self-care and activities of daily living, such as washing one’s hands to the point of pain, skin breakdown, or infection. Subtypes of OCD are often classified by the nature of the obsessional thoughts, such as contamination obsessions with cleaning compulsions, waste-related obsessions with hoarding compulsions, symmetry obsessions with ordering compulsions, religious obsessions with ritual-based compulsions, harm obsessions with checking compulsions, and ethical obsessions with reassurance-seeking compulsions. Some patients may experience obsessional thoughts without visible compulsions, such as intrusive thoughts of a violent or sexual nature or moralistic concerns. Newer theories suggest that compulsions may actually *precede* and trigger anxiety or obsessions (3) although by all accounts the cycle of obsessions and compulsions are hallmark features of OCD regardless of the order of occurrence.

Both OCD and TS are accompanied by a feeling of discomfort that precedes the behavior. In OCD the discomfort is emotional – anxiety related – whereas in TS the discomfort is a physical or sensory premonitory urge. Both conditions share the drive to engage in repetitive behavior; however, tics are classically considered involuntary and able to be suppressed only with effort whereas compulsions require higher order cognitive volition and awareness. The neurocircuitry behind TS and OCD share commonalities. As widely reported in the literature (4) the cortico-striatal-thalamo-cortical (CSTC) pathway is involved in both TS and OCD. The specific brain regions thought to be involved in this pathway include the Ventromedial Prefrontal Cortex, Anterior Cingulate Cortex, Orbitofrontal Cortex, and Parietal and Somatosensory Cortex. These are regions that are often associated with “action selection, performance, monitoring, response inhibition, and goal-directed behaviors” (3), behaviors often implicated in both TS and OCD.

Patients with TOCD present with overlapping symptomatology (Table 1). Unlike in OCD, patients with TOCD rarely describe obsessional thoughts but rather a feeling of intense physical discomfort, more akin to tic disorders, that drives compulsive behaviors. However, although this sensation is not initially driven by anxiety, it can become intolerable and anxiety provoking if not mitigated by engaging in the desired behavior. Similarly, the compulsions of TOCD have components of both OCD and TS. Movements are typically complex rather than simple tics and may involve tapping or touching things in a specific way, vocalizing phrases rather than simple sounds, or a multistep progression of movements, for example, a sequence of several different hand or body movements, rather than a single motor movement. Patients with TOCD often need to repeat the behaviors a number of times until they feel “right” and the discomforting premonitory sensation has passed. In this way, TOCD is both an externalizing and an internalizing disorder. It conflates the externalizing disinhibited movements of TS in parallel with internalized distress reminiscent of OCD when the behaviors are not completed “just so.” These behaviors have been termed “impulsions,” rather than compulsions, to help denote their particular phenomenology (5). While impulsions share some common features with compulsions, impulsions tend not to be driven by classic anxiety and so are not goal directed like classic OCD compulsions.

**TABLE 1:** Comparative characteristics of OCD, Tourette’s syndrome, and Tourettic OCD.

	OCD	Tourette’s syndrome	Tourettic OCD
Age of onset	11–15 years	Age 6–9 years	Age 7–13
Heritability	37–47% (95)	58–77%	Unknown
Prevalence	1–3% (96–98)	∼0.85–1% (4)	Unknown
Symptom course	Waxing and waning throughout life, typically persists if untreated	60–75% resolution by adulthood	Unknown
Symptom characteristics	Internalizing	Externalizing	Both
Precipitating cause	Provoked by intrusive anxious thoughts	Provoked by somatic sensations/premonitory urge	Provoked by somatic premonitory urge often with an associated cognition of something being “Not right” or needing to be “just so”
Content of behaviors	Repetitive behaviors, often involving volitional multi-step compulsions	Repetitive motor movements typically involving one muscle group or body part	Repetitive complex motor movements often with several steps including tapping, arranging, adhering to certain numbers of repetitions
Consequences	Anxious thoughts are briefly alleviated	Premonitory urge is briefly alleviated	Premonitory urge is briefly alleviated if tics are performed “just right”
Suppressable?	Yes, with effort	Yes, with effort	More difficult to suppress than OCD or TD alone
Behavioral therapy	ERP, CBT	CBIT	ERP/CBIT/CBT

The lack of clarity over the etiology of the patient’s discomfort as well as the nature of the repetitive behavior can cloud diagnostic clarification and treatment decisions and patients can be miscategorized as having only a tic disorder or only OCD. Categorization is particularly complicated and relevant as the treatment for tic disorders differs widely from that of OCD. Additionally, children can have comorbid classic tics and OCD, known as tic-related OCD, but may not possess the unique blend of symptoms required for TOCD; this can further confound the diagnosis of TOCD which is a clinical diagnosis. While many mental health disorders are clinical diagnoses, the unique challenges in TOCD are that the diagnostic entity itself is not as well-known as tics or OCD alone, and TOCD does not have clear diagnostic parameters in DSM-5 as do tic- and obsessive-compulsive related disorders. Therefore, the diagnosis of TOCD may be overlooked even by psychiatrists and neurologists.

The medical literature that evaluates tic-related OCD is also muddied; it can be difficult to interpret the nature of the population being studied as TOCD may not be explicitly distinguished within the study population. Many of the studies included in this article address tic-related OCD and we have attempted to extrapolate from this data to the extent possible. Focused studies on TOCD, of which there are currently few, are needed.

### Epidemiology, genetics, and epigenetics

Obsessive compulsive disorder presents in a bimodal distribution, with the first mean age of onset around 9–10 years (SD ± 2.5 years) and a second wave of new cases with mean onset in the early 20’s (6, 7). In contrast to OCD alone, tic-related OCD is characterized by earlier, pre-pubertal age of onset, and male predominance. Individuals with tic-related OCD have specific symptom clusters such as lower tendency toward compulsions related to checking, cleaning, and reassurance seeking and higher tendency toward compulsions such as rubbing, tapping, or touching associated with symmetry concerns or thoughts of exactness (8). The studies in tic-related OCD have shown mixed results in youth, with some reporting that tic-related OCD may present with intrusive thoughts of a violent or sexual nature that are not accompanied by a particular compulsion (9, 10). Other groups showed that individuals with tic-related OCD were more likely to experience washing and cleaning compulsions, hoarding, and ordering (11, 12).

### Genetics

Tourette syndrome is inherited in 70–80% of cases (13) making it one of the most heritable childhood-onset neuropsychiatric disorders (14), which has been widely reproduced in the literature (15, 16). Various approaches have been undertaken to evaluate the genetic architecture of TS including candidate gene studies, segregation analysis, linkage analysis, cytogenetics, copy number variants (CNV), studies of rare variations, genome-wide association studies (GWAS), and whole exome sequencing (WES). The Tourette Syndrome Association International Consortium for Genetics (TSAICG) and others investigated various susceptibility genes in the dopaminergic, serotonergic and glutamatergic pathways, such as the receptors and transporters *DRD2*, *DAT1* (*SLC6A3*), *HTR2A*, and *EAAT1* but later switched to GWAS and CNV studies (16). TSAICG contributed to the creation of the Psychiatric Genomics Consortium for Tourette Syndrome, which undertook the first genome-wide association study (GWAS) of TS. It found that no single nucleotide polymorphisms (SNP) reached genome-wide significance, but the top-ranking variants were enriched for genes that affect gene expression and methylation in the fronto-striatal circuitry, which is in line with current working models of TS neurocircuitry (17). A similar study done by the Tourette International Collaborative Genetics Study (TIC GENETICS), analyzed WES in families with TS and found that histaminergic pathway genes were highly enriched in TS etiology (L-histidine Decarboxylase- *HDC* enzyme that converts L-histidine to histamine) (18–20), again in line with current models of TS pathophysiology, which involve neurotransmission, inflammation, and smooth muscle tone.

In parallel, the genetic architecture of OCD has been analyzed by other large multicenter collaborations including the International Obsessive-Compulsive Disorder Foundation Genetics Collaborative (IOCDF-GC) and the OCD Collaborative Genetics Association Study (OCGAS). The meta-analysis from the two consortia of 2,688 individuals with OCD and 7,037 matched controls found that no SNPs reached genome-wide significance (21) but several glutaminergic system genes have been implicated (e.g., *GRID2*, *DLGAP1*). Genome-wide association studies (GWAS) have estimated the genetic heritability for OCD at approximately 0.37 in adults with OCD and 0.43 in childhood onset OCD (22). The age of onset of OCD is strongly linked to familial genetic loading. In pediatric OCD, individuals have a two-fold higher risk of having a first-degree relative with OCD as compared to adult-onset cases. The current understanding of the genetics of OCD has been extensively reviewed in the literature (23).

Previous data suggests that OCD and TS share some genetic blueprint. Among pediatric patients with OCD, greater than 50% exhibit tics. Likewise, an estimated 30–60% of patients with TS manifest symptoms of OCD (24). Patients with TS or OCD are also more likely to have a first-degree relative with either of these disorders (25). Additionally, biological relatives of probands with TS are more likely to develop OCD as compared to adoptive relatives of the same probands (26). This cross-disorder prevalence both within patients and within families suggests shared genetic underpinnings. Genome-wide associations in a combined sample of OCD and TS patients did not show overlapping polygenic scores for both disorders (27) although a genetic correlation between TS and OCD was estimated at 0.41 using genome wide complex trait analysis (22). These varying studies suggest a complex genetic background to each disorder, with small cumulative contributions from multiple genetic loci. Genetic studies in monozygotic twins show only 50% concordance rates in tic-related OCD/TOCD (28) highlighting the complex nature of heritability in these disorders.

The genetic architecture for TOCD is still unknown. Given that TOCD manifests with components of both TS and OCD, it is postulated to share genetic similarities to both disorders. Coffey et al. suggest that TOCD is more genetically similar to TS than to OCD because patients with TS and TOCD have higher rates of comorbid ADHD compared to patients with OCD (29). Ironically, newer advent technologies, designed to shed light on these questions, have in fact made the data even more disparate as the increased numbers of genetic studies yield conflicting results. Given the lack of clarity in both OCD and TS, it is no surprise that even less is known about TOCD. By extrapolation we presume that genes in the dopaminergic, serotonergic, glutaminergic and histaminergic pathways along the CTSC circuit are implicated. Epigenetic factors likely also contribute to variability in clinical presentation including age of onset, symptom severity, and symptom characteristics, although large population studies are needed.

### Environmental factors

It is widely known in the literature that environmental factors exacerbate tic/OCD symptoms. In addition to the known tendency of environmental stressors to exacerbate existing symptoms within an individual, prospective studies are elucidating a potential link between environmental factors and the onset of tics or OCD in individuals with no personal prior diagnosis but with a first degree relative with a CTD (30). While multiple environmental stressors are being considered, three in particular have been elucidated: (1) infection or inflammation, (2) perinatal complications, and (3) chronic childhood psychosocial stress.

### Infectious or inflammatory processes

A variety of neuropsychiatric disorders that include pre-existing tic, OCD, and TOCD symptoms are known to increase during infectious or inflammatory processes, suggesting immune-mediated mechanisms for these symptoms (31). Not only has active inflammation been shown in the neurocircuits underlying OCD symptoms (and by extension, the neurocircuitry of tics as these are overlapping networks in the brain) (32), but even a history of infection has been implicated in increased incidence of mental health disorders that include tics and OCD. A Danish study of over one million youth aged birth-17 years evaluated patient records in a period of up to 17 years following documented infection and found that a remote history of both streptococcal and non-streptococcal infection increased the risk of both tic and OCD symptoms (33). Interestingly, streptococcal infection in particular was linked to later development of tic disorders, while OCD arose from all-cause infectious etiologies (33).

The possibility of a specific connection between streptococcal infection and tic/OCD symptoms has been widely debated in the literature. While some studies have shown a link, others have failed to corroborate these results. A very recent European study did not confirm this specific association between tics and streptococcal infection (34). The prospective European Multicentre Tics in Children Study (EMTICS) across 16 European centers followed a cohort of 259 children aged 3–10 years with no prior history of tics but a first degree relative with CTD, to assess the presence of Group A Streptococcal (GAS) infection using throat swabs, serum Anti-streptolysin O titers (ASOT) and Anti-DNAse B (ADB) titers whether they had pharyngitis symptoms or not. Sixty-one children (23.6%) had new onset tics over a 1-year follow up with a strong association with male sex, but no statistical correlation linked those who developed tics with evidence of a prior GAS infection. Based on what is known about genetic heritability of tic disorders, the 23.6% of children who developed tics would fall within the expected range based on genetic heritability alone, suggesting little contribution from a prior GAS-related etiology.

The hotly debated connection between GAS infection and tic/OCD symptoms is disputed not only as it relates to symptom flares in diagnosed patients, but also the potential for GAS to trigger abrupt onset CTD or OCD in previously healthy children. The diagnostic term PANDAS – Pediatric Autoimmune Neuropsychiatric Disorders Associated with Streptococcus – was introduced in 1998 to describe a presumed subset of acute onset OCD and tic disorders that occur spontaneously in response to GAS infections, primarily in children (35, 36). This relationship has since been expanded to include non-streptococcal infections as well, under the umbrella term PANS – Pediatric Acute-onset Neuropsychiatric Syndrome, a fairly vague and inclusive term that may have led to high rates of over-diagnosis, although a small subset of true cases is probable. One of the key diagnostic criteria for both PANS and PANDAS is the abrupt onset of OCD and/or tic-like behaviors. A suggested mechanism is cross reactivity of anti-strep or other antigen-specific antibodies that affect neural circuits in the basal ganglia, a structure implicated in the neurocircuitry of both tics and OCD (37). However, the evidence to support this mechanism is insufficient as no specific antigen-antibody interactions have been experimentally demonstrated in cases of PANDAS, nor have specific biomarkers been elucidated. Instead, the proposed mechanism is borrowed from what is known from Sydenham’s chorea, a similar disorder that is precipitated by GAS infection and associated with basal ganglia dysfunction (37) and for which antigen-antibody interactions in the basal ganglia have been demonstrated. Both PANDAS and PANS remain widely debated in the literature because it is often unclear if pre-existing mild tic or OCD symptoms were escalated by infection to the level of clinical concern, or whether the symptoms are truly abrupt onset and directly caused by infectious processes. To the extent to which infection either exacerbates or gives rise to abrupt onset tic or OCD symptoms, TOCD is likely equally exacerbated by this phenomenon, and the complex and atypical tics described in the literature may indicate TOCD symptoms.

In a timely example of the effects of infections, both tic and OCD symptoms have been shown to sometimes increase in relation to infection with severe acute respiratory syndrome coronavirus 2 (SARS-CoV-2), the pathogen behind COVID-19. This increase is thought to be related to both the infectious agent itself as well as to increased environmental stress of quarantining and the effect of social protocols that may encourage compulsions such as cleaning, checking, and hand washing (38).

### Perinatal complications

A 2016 study examining 1,113 patients from the Tourette International Collaborative Genetics Study evaluated 586 patients with CTD and 527 healthy family controls and found that pre- and perinatal complications result in increased incidence of CTD and OCD (39). These complications included premature birth (OR = 1.72), severe hyperemesis gravidarum (OR = 2.57), and problems during delivery (OR = 1.49), suggesting that early adverse events predispose to later development of tics and OCD, and by extrapolation likely to TOCD. Interestingly, prenatal complications were more closely associated with development of CTD while problems during delivery and immediately post-natal were more closely linked to OCD. A parallel similar study comparing perinatal history of 130 youths with OCD compared to 49 age-matched controls found that history of maternal prenatal illness and higher rates of difficulties during labor such as induction, use of forceps, prolonged labor, and conversion to Cesarean-section were correlated with earlier age of OCD, greater symptoms severity, and higher incidence of comorbid tic disorders/TOCD (40). Similar studies have reinforced the increased incidence of tic-related OCD vs. TOCD in cases of prenatal and perinatal complications (41). The increased incidence of early adverse events suggests environmental or epigenetic contributions that may occur during key critical periods of pre- and perinatal neuronal development. Much remains to be understood regarding the timing of injury as it relates to downstream development of CTD or OCD. The discrepancy in the timing of pre- vs. post-natal injury correlating to later onset CTD vs. OCD suggests that CTD, OCD, and TOCD may lie along a developmental spectrum, such that proximal injury at different stages of development affects distal symptom characteristics.

### Psychosocial stress

Psychosocial stress has been linked to the development of CTD and OCD as well as to symptom severity, suggesting that the same is true in TOCD patients (42–44). Lin et al. demonstrated that children with OCD scored significantly higher on self- and parent-rating scales of perceived stress, and that higher levels of stress correlated with later symptom severity as well as later depressive episodes (45). Similarly, in a survey completed by patients with TS, 96.8% of patients identified psychosocial stressors as a major precipitant for tic symptom severity (46). This correlation has been exemplified by the stressors endured during the recent COVID-19 pandemic, which was shown to have increased OCD severity across all symptom dimensions (43). In a population of patients with OCD, over 60% reported at least one stressful life event prior to the onset of symptoms, and of those over 1/3 rated the stressful event as severe (i.e., death of a family member, major illness, etc.) (42). Other studies suggest that children and adolescents with TS and OCD tend to experience significantly more psychosocial stress than children without these conditions, including a higher number of daily stressful events as well as major life stressors of a chronic nature (47).

### Limitations of current knowledge

Taken together, genetic, epigenetic, and environmental factors play a role in driving both tic and OCD symptoms as described above. The strong familial tendency for first degree relatives with a shared family history to develop *either* OCD or CTD, confirms that these disorders do not follow simple inheritance patterns, nor are they likely to be exclusively determined by genetic factors. Clinically, we have observed that CTD are prevalent among patients with a parent with CTD *or* OCD and vice versa, as compared to the general population. Likewise, it is common for one sibling within the family to develop a CTD, while another sibling may develop OCD suggesting epigenetic or environmental contributions to an underlying genetic predisposition. Unfortunately, no definitive data has yet elucidated why one family member may develop a CTD while another may develop OCD or TOCD. This remains an important and outstanding question that requires further study.

## Neurocircuitry

The overlapping symptomatology of OCD and TS suggests similar mechanisms for reduced cognitive control over motor and behavioral inhibition. Therefore, it is not surprising that ADHD and ASD run comorbid with TOCD because ADHD and ASD both arguably manifest with difficulties of inhibition indicated by impulsivity (ADHD) and repetitive, stereotyped behaviors (ASD).

The neurocircuitry of tic disorders and OCD are among the best characterized within neuropsychiatric disorders. In recent years, studies have implicated up to five circuits that play critical roles in the neurocircuitry of OCD and may explain some of the heterogeneity of this disorder (3, 48). Multiple neuroanatomical regions have been implicated including the amygdala and hippocampus (49), as well as frontoparietal and cerebellar structures (48, 50). However, the most heavily implicated and replicated region on fMRI imaging of both tic and OCD disorders is the cortico-striatal-thalamo-cortical (CSTC) loop. This network connects the prefrontal cortex (PFC), basal ganglia (including the caudate nucleus, putamen, nucleus accumbens, internal and external segments of the globus pallidus, subthalamic nuclei, and substantia nigra), and the thalamus, then returning to the PFC. Multiple reports of disruption to these networks, such as due to trauma or infection of the PFC or basal ganglia result in OCD and tic behaviors (35, 51–53).

The CSTC circuit is comprised of three primary sub-loops as described below. A full description of these circuits can be found in the medical literature (3, 48).

1. The *Motor Circuit* connects the sensorimotor and premotor cortices to the basal ganglia via the posterior-lateral putamen which in turn projects to the globus pallidus externus (GPe), the globus pallidus internus (GPi), and the subthalamic nuclei (STN). The GPi outputs to the ventrolateral thalamus en route back to the cortex. This loop is involved in habit formation and top-down motor control.
2. The *Associative Circuit* (also known as the Dorsal and Ventral Cognitive Circuits) involves circuits from the dorsolateral prefrontal and lateral orbitofrontal cortices, which project through the caudate nucleus to the other basal ganglia structures described above. This circuit is thought to play a role in goal-directed behavior.
3. The *Limbic Circuit* involves the orbitofrontal and anterior cingulate cortices, which project to the caudate nucleus (CN), putamen, GPe, GPi, and STN and thalamus before returning to the cortex. This circuit is involved in motivation and reward.

Relative upregulation and downregulation of the CSTC network —the motor circuit, the associative circuit, and the limbic circuit —are postulated to underlie the neurophysiology of both tics and OCD. In both conditions, patients experience a loss of top-down control in which anxious stimuli or sensory perceptions lose their rational salience and become overly discomforting. This triggers repetitive behaviors by means of the associative circuit and basal ganglia activation, resulting in reward feedback that diminishes the anxious (intrusive thoughts) or somatic (premonitory urge) triggers. However, the relative *balance* of these loops has been postulated to differ between TS and OCD. While both symptom clusters involve the associative circuit, OCD is thought to more heavily involve the limbic-associative circuits (associated with anxious distress), while TS involves the motor-associative circuits (associated with a somatic premonitory urge) (54, 55). As Shephard et al. have pointed out, both tics and OCD involve an “intolerance of uncertainty… a tendency to perceive and interpret uncertain situations as negative or threatening” (56). Such intolerance leads individuals to develop motor or behavioral responses that minimize their anxiety or discomfort.

These findings have been supported by fMRI and Positron Emission Tomography (PET) in separate studies of patients with tics or OCD. In a study of four patients with OCD exposed to progressively more distressing triggers, McGuire et al. (57) correlated symptom intensity of obsessive thoughts and desire to perform compulsions with increased blood flow in the right inferior frontal gyrus, caudate nucleus, putamen, globus pallidus, thalamus, left hippocampus, and cingulate gyrus. Notably, the premotor cortex and sensorimotor cortex were not heavily implicated, in keeping with the notion that OCD relies more heavily on limbic-associative circuits. Analogously, fMRI imaging of 13 patients with TS with spontaneous tics compared to 21 healthy controls with volitional tic-like movements (58) corroborated previous findings of elevated activity in all portions of the motor pathway including the sensorimotor cortex and basal ganglia. This finding supports the notion that dysregulation of the motor-associative circuit causes a lack of control over tic behaviors. Furthermore, the severity of tic symptoms was also positively correlated with increased neural activity in the amygdala/hippocampus complex, and was heightened in TS patients with spontaneous tics as compared to voluntary “tics” among healthy controls. This suggests that these regions may be involved in generating the premonitory urges of CTD which distinguish them from volitional movements. In contrast, the TS group had weaker activity in the caudate and anterior cingulate cortex, which exert top-down control over motor pathways though may fail to do so in patients with CTD. Activity in these regions negatively correlated with increased tic severity (58).

Although further studies are required, preliminary data suggest that TOCD symptoms may arise from involvement of all three sub-loops as part of an “intermediate” neurocircuitry that lies along the “impulsive-compulsive spectrum” (59). Indeed, studies among a clinically heterogeneous population of TS patients have postulated that while simple tics are most closely associated with changes in the motor circuit, complex tics (more reminiscent of TOCD behaviors) are associated with changes in the associative circuit, and frank OC behaviors in patients with TS are associated with greater dysregulation of the limbic circuit (54, 55). Regional brain involvement of the associative and limbic circuits in patients with complex tics or OC behaviors is more similar to OCD neurocircuitry in which the limbic-associative circuits are more heavily implicated than in simple tics alone. This heightened dysregulation of the associative-limbic circuit, in addition to the motor circuit, may yield a loss of top-down executive control, resulting in an inability to rationally analyze the accuracy of one’s emotions, control their motor responses, or inhibit reward learning and habit formation. Phenotypically, the imbalanced network drives complex tic-like behaviors of a compulsive and partially anxious nature, commensurate with TOCD. Few neuroimaging data exists to confirm the specific circuits or neuroanatomical regions involved in TOCD specifically as compared to OCD and CTD, and further studies are warranted.

In an attempt to modulate the neurocircuitry for purposes of treatment, deep brain stimulation (DBS) has been used for adults with treatment-refractory Tourette syndrome (TS) since the late 1990s. Several different nuclei within the CSTC network have been explored as potential targets for DBS (60–62). Four targets within the basal ganglia have been most commonly used: the centromedian nucleus–nucleus ventrooralis internus complex of the thalamus (CM-Voi), the centromedian nucleus–parafascicular (CM-Pf) complex of the thalamus, the posteroventrolateral (pvIGPi), and the anteromedial portion of the globus pallidus internus (amGPi). A recent review on DBS in TS (63) analyzed 65 studies and included 376 patients. Overall, nearly 70% of the patients had >50 reduction on Yale Global Tic Severity Scale (YGTSS) scores regardless of these different targets. Interestingly, in tic patients with comorbid OCD, DBS in CM-Pf nucleus resulted in a reduction in OCD symptoms as measured by the Yale Brown Obsessive Compulsive Scale (Y-BOCS) scores. For treatment-refractory OCD, the most widely used DBS targets have been the nucleus accumbens and the anterior limb of the internal capsule (NA/ALIC) (64–66). DBS targeting NA/ALIC has also been successfully used in patients with TS (67) again proving the structural and functional interconnectivity between these two disorders (67, 68). DBS therapy is invasive, may have side effects and has not been approved for use in the pediatric population. In addition, the optimal targets for symptom control may need to be individualized. Future efforts of combining fMRI with stereotactic surgery may help determine the best targets for patients with TOCD (64–66, 69).

## Pharmacological treatment

Unfortunately, as described above, TOCD is not independently classified within the DSM-5. It is best but inadequately captured under the diagnostic specifier of “tic-related OCD,” defined as an OCD “diagnostic subtype based on whether the individual has a past or current tic disorder.” As a result, pharmacological treatment for these patients may mistakenly concentrate on medication specific solely to OCD, which is often inadequate for remission of TOCD symptoms. To better account for TOCD holistically when considering the best treatment, clinicians should consider their condition as a case of comorbid OCD and tics disorder rather than a tic-related subtype of OCD (1).

**FIGURE 1:**
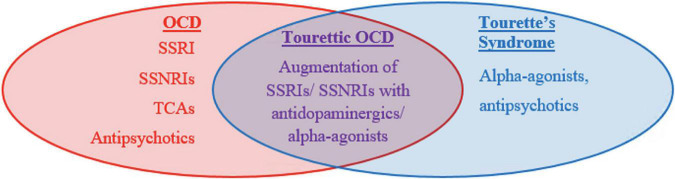
Similarities and differences in the pharmacologic management of tic disorders and OCD.

First line agents differ between CTD and OCD (Figure 1). OCD and anxiety spectrum disorders are treated with strong serotonergic agents such as Selective Serotonin Reuptake Inhibitors (SSRIs) or Selective Serotonin and Norepinephrine Reuptake inhibitors (SSNRIs) (70). These agents work by blocking serotonin reuptake and therefore increasing serotonin availability at the synapse. Four SSRIs: fluvoxamine, paroxetine, fluoxetine, and sertraline, are FDA approved for the treatment of OCD in adults and may be utilized in children. These medications can address the somatic experience of anxiety including headache, fatigue, muscle pain, and GI upset in addition to treating mental anxiety. Tricyclic antidepressants (TCAs), which increase the availability of all monoamines including serotonin, norepinephrine, and dopamine, are equally effective though are less favored due to higher side effect profile compared to SSRIs. Side effects include dry mouth, blurry vision, constipation, and fatigue among others. One TCA, clomipramine, has been FDA approved to treat OCD. 40–60% of patients do not remit with SSRIs alone and have been shown to benefit from augmentation with antipsychotics. Either first- or second-generation antipsychotics are considered highly effective but are avoided as first line agents due to the high side effect profile including metabolic side effects, cognitive dulling, sedation, and extra-pyramidal side effects. Common antipsychotics used for this purpose include haloperidol (first generation), aripiprazole (second generation), or risperidone (second generation). Augmentation is implemented if SSRIs or TCAs fail to show response following 3 months of therapy at a therapeutic dose. As a general rule, “failure to respond” is defined as less than a 25–35% reduction on the Child Yale Brown Obsessive Compulsive Scale (71).

**FIGURE 2:**
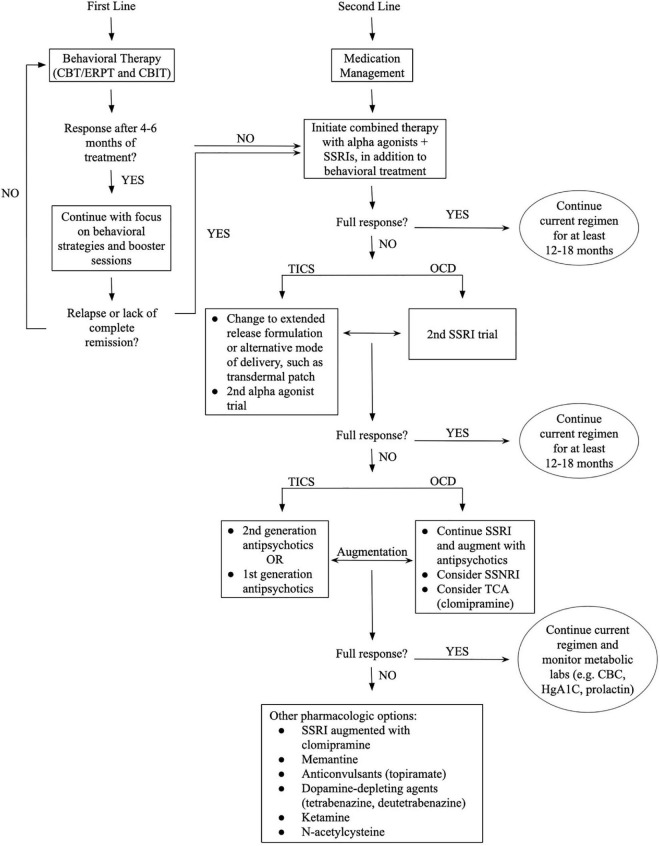
Proposed treatment algorithm for patients with TOCD.

Like with OCD, tic disorders are felt to respond well to antipsychotic medications but have high risk of side effects. Two in particular, haloperidol and pimozide, are FDA approved for this purpose but are typically deferred until first line agents, the alpha agonists, have been tried. Alpha agonists, such as clonidine and guanfacine, are considered first line agents for tic disorders including Tourette syndrome and act by stimulating the post-synaptic alpha-2A receptors in prefrontal cortical pyramidal cells which may stimulates the frontal cortex to regulate attention and thus suppress tics. They may also decrease arousal. Although generally recognized as less effective than antipsychotic medications, they are favored as first line agents due to their lower side effect profile which can include hypotension and sedation. Children with TOCD often require combined treatment due to their overlapping symptomatology. They are more likely to require polypharmacy and higher dosing regimens (Figure 2).

A previous meta-analysis reviewing 15 clinical studies noted that tic symptoms in OCD patients are a substantial indicator of treatment-refractory response following SSRI monotherapy and that augmentation of SSRIs with antipsychotics allows for more sustained improvement in patients with comorbid TS and OCD (72, 73). Similarly, among tic-related OCD patients who were treatment refractory to a 12-week SSRI trial, addition of either risperidone or aripiprazole to an SSRI improved OCD symptoms in 56.5% of patients, tic symptoms in 68.1% of patients, and both OCD and tic symptoms in 50% of patients, with high likelihood of capturing TOCD patients within this population (74). One possible mechanism behind the SSRI-antipsychotic co-administration suggests that the SSRI targets OCD symptoms, while antipsychotics reduce tic symptomology in tic-related OCD and TOCD patients. Equally likely is that TOCD (and likely CTD and OCD) involves a combination of serotonergic and dopaminergic signaling, such that conjoint SSRI-antipsychotic administration modulates both of these neurotransmitter pathways and either treatment alone is insufficient. Since SSRI monotherapy generally serves as the first-line treatment in OCD patients, and antipsychotics are among the primary treatments for TS, adjunctive therapy with both medications allows for more effective pharmacological treatment of TOCD (Figure 1).

In addition, pharmacologic treatment of comorbid ADHD with alpha-agonists, atomoxetine or stimulants can help control the disinhibition and impulsivity that is common with TOCD patients. Historically, stimulants have been avoided in patients with tics; however, a recent Cochrane review analyzing eight studies with 510 participants with comorbid tics and ADHD discredited this cause of concern. It showed that symptoms of ADHD and tics, including impulsivity, actually improved when several stimulant and non-stimulant medications were used, including methylphenidate, guanfacine, clonidine, and a combination of methylphenidate and clonidine. The single exception to this was one study of 3 weeks duration that found exacerbation of tics when using high dose dextroamphetamine (75). As always, an individualized approach to the choice of medication, means of delivery (short release vs. extended release vs. patch) and dose adjustment should be taken into consideration.

First line agents differ between these disorders. OCD is treated with SSRIs, SSNRIs, and less commonly with TCAs as first line agents. CTD are treated initially with alpha agonists. In both disorders, antipsychotics can be used for augmentation or as primary agents for children who do not respond or cannot tolerate first line agents. TOCD often warrants combined treatment as well as higher dosing regimens due to overlapping symptomatology. Management with antipsychotics is required more often as compared to CTD or OCD alone.

## Behavioral treatment modalities for patients with Tourettic obsessive compulsive disorder

Various psychological and behavioral therapies are used as monotherapy or in conjunction with pharmacologic therapy for OCD or TS depending on the patient’s underlying symptoms (Figure 2). Cognitive Behavioral Therapy (CBT) and Exposure-Response Prevention Therapy (ERPT) are widely accepted as first-line behavioral interventions for OCD related symptoms, while Comprehensive Behavioral Intervention for Tics (CBIT) is a widely accepted behavioral treatment for tic disorders. The American Academy of Neurology 2019 Practice guidelines support CBIT as the first line treatment for tics, preceding medication (76). Each of the therapy modalities described here have been shown to be highly effective even in the absence of medication management, although a major barrier is the lack of access to trained providers.

The focus of these therapies differs based on the identified disorder and the symptoms being treated. CBT and ERPT start by identifying obsessional thinking patterns, and slowly train a patient to tolerate increasing levels of anxious distress while suppressing the desired compulsions. CBIT is arguably more behaviorally focused on learning to recognize premonitory urges and the occurrence of tics which are often out of a patient’s conscious awareness. It considers the circumstances that trigger tics and seeks ways of diminishing these triggers. The emphasis is on finding the best competing response for each individual tic, focusing on one discrete tic at a time. Unfortunately, because tic disorders and OCD are often conceived of as distinct diagnoses, patients are often referred to only one therapeutic modality.

Utilizing behavioral therapy in addition to medication management has been shown to have higher rates of symptom remission in tics and in OCD alone (77). CBIT was shown to be efficacious for youth aged 9–17 years with CTD or TS (78). In a combined population of children with tic-related OCD, The Pediatric OCD Treatment Study (POTS II) (79) examined the efficacy of CBT augmentation strategies for youth who had only partially responded to SSRI treatment. Those receiving combination therapy in the form of medication management *and* traditional Cognitive Behavioral Therapy (CBT) had significantly greater reduction of OCD symptoms compared to medication alone (ES = 0.85). Because TOCD is even more nuanced than tic-related OCD, not only can its pharmacologic management be challenging but also the type of behavioral therapy employed. Similar to the need for combined medication management of both tic and OCD symptoms, we postulate and have found in our own clinical practice that TOCD responds best to adjunctive behavioral interventions targeting *both* tic and OCD symptoms. In light of the complex neurobehavioral presentation of TOCD, outcomes are significantly improved by focusing on the anxious-somatic distress of OCD through either CBT or ERPT in addition to targeted behavioral interventions for tics using CBIT. The emphasis on unhelpful thinking styles in CBT or ERPT can decrease the anxiety that triggers tics and repetitive behaviors and help develop strategies for coping with the debilitating nature of TOCD (80). In parallel, CBIT may help train the patient to perform a competing behavior when they feel the urge to tic, which over time lessens the premonitory urge.

To date, no singular behavioral monotherapy exists exclusively targeted to TOCD. A re-imagined version of CBT, ERPT, or CBIT could address the unique nature of TOCD including the anxious-somatic premonitory sense that precedes TOCD impulsions. For example, clinicians trained in both CBT for OCD (77) and CBIT may be able to blend these therapies to expose patients to core obsessional fears, as in CBT, while using competing responses to target the tic component of the symptoms, as in CBIT (81). Similarly, a classic tool used in ERPT is the fear hierarchy ladder, in which patients identify increasingly stressful situations in ascending order from least stressful to most stressful and then work to mitigate their anxiety and suppress compulsions while tolerating increased levels of exposure to the identified stressor. In a TOCD-specific version of this ladder, therapists might help patients learn to tolerate their tics, first by continuing to engage in their tics without repeating them “just so” (i.e., tolerate doing the tics “imperfectly”), which could then progress higher up the ladder to full suppression of tics altogether.

Behavioral therapies are suggested to be the first line of treatment and medication management can be introduced as a second line treatment though medications are often required. Depending on the response to the initial intervention the next steps are outlined from the top to bottom. Patients with TOCD require treatment of both tic and OCD symptoms in parallel, typically requiring polypharmacy as well as behavioral interventions of more than one modality. Monitoring of both tic and OCD symptoms is essential as patients may have improvement in one domain while having ongoing symptoms in the other. This is denoted above by the bidirectional arrows to indicate the need for attention to both sets of symptom clusters which may be independent or wax and wane in parallel. Of note, while the first line agents for these disorders differ, there is overlap in second line agents which can be useful. Specifically, first or second-generation antipsychotics are helpful second line agents for both tic and OCD symptoms and can be used as monotherapy or as augmentation to SSRIs or alpha agonists. Similarly, SSRIs, which are first line for OCD, can also be added as second line agents to augment alpha agonists or antipsychotics for management of tics. In reality, the “full response” is rarely encountered, and an individualized approach to treatment has to be taken.

## Special considerations in the pediatric population

Tourettic OCD symptoms often initially present with tics around age 7–9 and later evolve into more complex tics with obsessional features as children approach adolescence. These are very important years for a child’s psychosocial development. For most children, the peak of TOCD symptoms coincides with middle school, when patients are often more aware of their difficulties and their presentation has stronger implications for social, emotional, and academic functioning.

At home, the family may be affected in multiple ways. Many parents of children with OCD report irritability in their children (82) that affects family dynamics. Parents may accommodate the TOCD behaviors in order to avoid frustrations or tantrums (12), or may lack insight and try to rationalize or minimize their child’s behavior, and provide frequent reassurance which then enables the behavior and may delay treatment (80). Some situations in which the child needs to perform their ritualized behaviors “just right,” may involve other family members who are asked to participate in a sequence of behaviors that can interfere with family schedules and lead to frustration and distress by all involved (83). The parents’ own mounting frustration and anxiety is often projected onto patients with TOCD, creating a difficult and vicious behavioral cycle. Moreover, the treatment-resistant nature of TOCD as well as polypharmacy with various potential side effects exacerbates distress in both patients and parents.

At school, behaviors may interfere with academic functioning or lead to disciplinary action, especially when tics involve yelling of inappropriate phrases, complex movements during class, or behaviors misinterpreted as aggression such as throwing objects or exposing parts of one’s body to peers (such as pulling down one’s pants). The risk of bullying, social anxiety and fear of being different from peers can be very real. Children with TOCD often work hard to suppress tics at school to the extent possible, although this may cause them to avoid social activities, become temperamentally more withdrawn, and in some cases worsen anxiety, which has the paradoxical effect of exacerbating their behaviors. Friendships and age-appropriate social development are impeded. Shame and embarrassment may cause children to become withdrawn and decline to share their struggles with parents and providers, exacerbating academic decline and delaying treatment. Children with tics/OCD/TOCD are at increased risk of developing additional disorders such as ADHD, anxiety or depression, which further impede social, emotional, and academic development (84).

**FIGURE 3:**
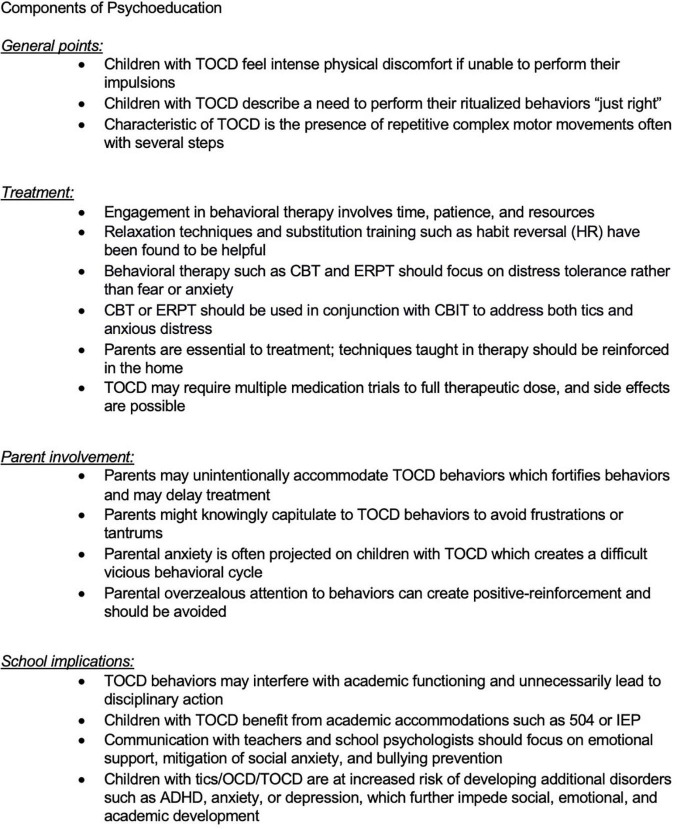
Components of psychoeducation for children with TOCD.

It is important for the medical professionals taking care of children with tics, OCD, or TOCD to provide them not only with the right pharmacotherapy and psychotherapy but to provide support for the school and family settings. Family guidance, individual therapy for the child, family therapy (taking into account the needs of siblings as well), adequate contact with the school nurse, psychologist, teachers, and coaches can be very beneficial (Figure 3).

For patients with TOCD, it is important to encourage physical activity rather than the use of electronics given the known worsening effect of screen time on the prevalence of tics (85). Vigorous physical activity for at least 1–2 h every day is encouraged as there may be some benefit for patients with tics and/or compulsions, making it a promising tool to reduce symptoms as an adjunct to medication and behavioral interventions though the full extent of benefit is not entirely clear (86–88). Exercise is also known, however, to be protective against development of anxiety disorders as well as to significantly reduce anxiety among sufferers (89). The combined effect on tics, compulsions, and anxiety suggests benefit for TOCD. Some examples of activities that can be helpful include swimming, skating, riding a bicycle, soccer, and long-distance running.

In addition, there is evidence that sufficient sleep and a good sleep-wake cycle can decrease anxiety and other mood-related comorbidities (90). We therefore encourage adherence to a good sleep schedule and we generally discourage the intake of caffeine in adolescent patients as it may interfere with their sleep-wake cycle. There are currently no clear dietary recommendations for patients with tics or OCD, or for that matter TOCD, except one small pilot study suggesting some benefits from a gluten-free diet for children with tics and comorbid OCD (91). Further research is currently being done on the effect of diet on CTD, OCD, and TOCD.

Tourettic OCD is less commonly recognized as a diagnostic entity compared to OCD or tics alone and often necessitates a broad treatment approach and psychoeducation for all involved. Even some mental health clinicians may not be familiar with the unique treatment needs of TOCD. Medication management typically requires polypharmacy, while therapy may require more than one modality such as Exposure Response Prevention Therapy (ERPT) for OCD with concurrent Cognitive Behavioral Intervention for Tics (CBIT). Close communication between parents, clinicians, and schools is beneficial. Furthermore, educating peers, neighbors, coaches, and even non-mental health clinicians who may be caring for the child can be illuminating and may decrease ostracization, bullying, and can increase a child’s functioning by providing a more supportive environment.

## Discussion

Since the initial characterization of TOCD (1) there has been a better appreciation of this particular subtype of patients. Given the lack of precise diagnostic parameters, however, very few studies specifically address this population. Thus, prudence is warranted when discussing TOCD or extrapolating from the existing literature on TS and tic-related OCD. Many studies address tic-related OCD, and while patients with TOCD are likely broadly included in this category, the specific nature of TOCD is narrower and patients’ symptoms are more intertwined. It is important that TOCD be distinguished as a separate, well-defined syndrome because these patients often require different management than other patients on the tic and OCD spectrum. Our current understanding of TOCD supports an endophenotype that shares commonalities with TS and OCD in the brain CSTC circuitry as well as genetic traits, and yet is unique and even more complex than TS or OCD alone.

Tourettic OCD has a later age of onset than classic tic disorders although tics may be the first presentation of TOCD prior to the onset of OCD features. Part of the challenge with TOCD is that symptoms not only wax and wane over time, similar to OCD and TS flares, but that the central nature of the behaviors also evolves over time. Specifically, patients may first present with classic simple tics which morph into complex tics, and later take on the impulsive-obsessional quality of complex repetitive movements that need to be done “just right.” For example, vocal tics may begin as a single vocalization that over time becomes a word, then a phrase, then eventually the need to repeat the phrase multiple times in a particular way. The later tendency to repeat complex behaviors in a specific way, although seemingly akin to compulsions, is distinguished from classic OCD in that the behaviors of TOCD are driven by a somatic urge or feeling of somatic distress rather than anxious, intrusive, obsessional thoughts. Behaviors characteristic of TOCD involve repetitive complex motor movements often with several steps, including tapping, arranging, adhering to certain numbers of repetitions. Patients report that their mixed behaviors are more difficult to suppress than OCD or TS alone.

This moving target of symptoms poses diagnostic challenges even to providers familiar with OCD and Tourette patients. The established way of conceptualizing mental health disorders is to put them into specific taxonomic systems (92), categorized as distinct neurological or psychiatric disorders based on clinical presentation. The Diagnostic and Statistical Manual of Mental Disorders (2) and the International Classification of Diseases (ICD) (93) are long-established and well respected diagnostic compendiums used for practical reasons to describe various psychiatric entities, and are based on distinguishing disorders from one another using clearly delineated symptom parameters.

**FIGURE 4:**
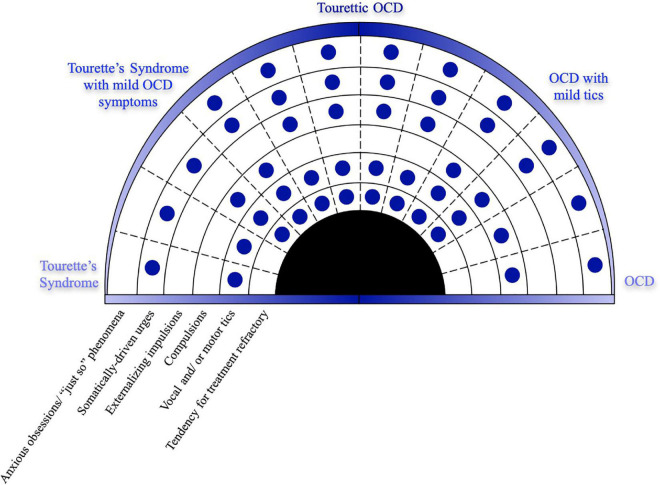
TOCD may represent a unique endophenotype along a clinical spectrum of OCD and tic disorders.

There is a newer trend, however, to think about these disorders as overlapping or spreading across the continuum of clinical presentations. The modern transdiagnostic approaches to neuropsychiatric disorders challenge researchers and providers to think about mental health disorders more broadly than categorizing them into specific symptoms and cause driven nosologies. The interplay between biological, behavioral, psychosocial and cultural processes are not limited to established diagnostic boundaries (94). Given the overlapping neurocircuitry, somewhat overlapping genetics, and high frequency of comorbidity, it is possible that tics and OCD exist within the same larger syndrome. We can conceptualize that tics and OCD lie on two ends of the same diagnostic spectrum on which TOCD can be found somewhere in the middle, sharing characteristics of both (Figure 4). Conceptualized slightly differently, given that TOCD tends to be treatment refractory, it may instead represent the severe end of the spectrum, with tic disorders and OCD existing along a developmental gradient of which TOCD is the most severe presentation. We proffer that TOCD deserves validation as a novel diagnostic entity within this continuum.

Transdiagnostic approaches help us describe and understand cases that share characteristics of both disorders and do not care if they fall into the specific realm of psychiatry or neurology. We are trying to describe processes within one brain comprised of millions of synaptic connections. These neuropsychiatric symptoms do not exist in discrete silos but rather may arise from overlapping neuropsychiatric circuits. Genetics plays an important role in defining the predisposition to OCD or tic behaviors, but epigenetic and environmental factors might determine the varying presentation in different individuals. Environmental factors potentially include dietary habits, the status of gut microbiome composition, physical exercise, or the type of behavioral conditioning that is present in various families. The individual’s socioeconomic status, demographics, family dynamics, access and initiation of pharmacotherapy and behavioral therapy might also contribute to the specific presentations.

This review suggests that TOCD exists on a continuum with TS and OCD that crosses the boundaries of each and presents with specific characteristics of both disorders. We propose that TOCD should be included in the next version of DSM as a specific, separate diagnosis that requires a multifaceted therapeutic approach. Our understanding of why some individuals present with isolated TS, others with tic-related OCD, while others develop TOCD, is still very limited. More research should be directed at understanding if any factors can be modulated in the developing brain, given that the multigenic background cannot be changed. Most patients will first present with waxing and waning motor and vocal tics, then subsequently develop OCD tendencies, and a subset of patients will develop TOCD. Perhaps earlier treatment with multiple pharmacologic agents to encourage synergistic effects might help the diagnostic spectrum not to progress too far. Behavioral therapy should extend beyond the boundaries of current techniques, including “customized” CBT, ERPT, and CBIT techniques. We hope that new research avenues will include detailed genetic analysis combined with imaging studies such as functional MRI or PET to elucidate the pathogenesis of these disorders. Collaborations between clinicians and researchers from diverse fields of expertise including psychiatry, neurology, psychology, genetics, and molecular biology would maximize recruitment of patients for large prospective observational studies and randomized controlled studies for interventions specifically addressing TOCD patients. Our hope is that increased awareness of this clinical entity will yield downstream interventions and quality of life improvements for those suffering from TOCD.

Tourettic OCD is a unique endophenotype that shares features of both Tourette Syndrome (on the left side of the diagram) and OCD (on the right side). The need to perform tics in a complex and precise way, known as the “just right phenomenon,” is the hallmark of TOCD and lies at the interface of the premonitory somatic urges of tics and the anxious obsessions of OCD. This diagram highlights the unique symptomatology of TOCD as it manifests symptoms of both CTD and OCD.

## Author contributions

TK and KT contributed equally to this research and writing of the manuscript, including research and new content, and share equal intellectual contribution to this document. JW and TB provided assistance with formatting, literature review, proofreading, creation of figures, and wrote selected sections of the document. GB assisted with research and formatting. All authors contributed to the article and approved the submitted version.

## Conflict of interest

The authors declare that the research was conducted in the absence of any commercial or financial relationships that could be construed as a potential conflict of interest.

## Publisher’s note

All claims expressed in this article are solely those of the authors and do not necessarily represent those of their affiliated organizations, or those of the publisher, the editors and the reviewers. Any product that may be evaluated in this article, or claim that may be made by its manufacturer, is not guaranteed or endorsed by the publisher.

## References

[1] Mansueto CS Keuler DJ. Tic or compulsion?: it’s Tourettic OCD. *Behav Modif.* (2005) 29:784–99. 10.1177/0145445505279261 16046664

[2] APA. *Diagnostic and Statistical Manual of Mental Disorders (Dsm-5^®^).* 5th ed. New York, NY: American Psychiatric Association (2013).

[3] Jahanshahi M Obeso I Rothwell JC Obeso JA. A fronto-striato-subthalamic-pallidal network for goal-directed and habitual inhibition. *Nat Rev Neurosci.* (2015) 16:719–32. 10.1038/nrn4038 26530468

[4] Robertson MM Eapen V Singer HS Martino D Scharf JM Paschou P Gilles de la Tourette syndrome. *Nat Rev Dis Primers.* (2017) 3:16097. 10.1038/nrdp.2016.97 28150698

[5] Flow Psychology. *Tourettic OCD.* (2017). Available online at: https://flowpsychology.com/Tourettic-Ocd (accessed April 22, 2022).

[6] Geller D Biederman J Faraone SV Frazier J Coffey BJ Kim G Clinical correlates of obsessive compulsive disorder in children and adolescents referred to specialized and non-specialized clinical settings. *Depress Anxiety.* (2000) 11:163–8. 10.1002/1520-6394200011:43.0.Co;2-3 10945136

[7] Rasmussen SA Eisen JL. The epidemiology and clinical features of obsessive compulsive disorder. *Psychiatr Clin North Am.* (1992) 15:743–58. 1461792

[8] Leckman JF Grice DE Boardman J Zhang H Vitale A Bondi C Symptoms of obsessive-compulsive disorder. *Am J Psychiatry.* (1997) 154:911–7. 10.1176/ajp.154.7.911 9210740

[9] Kalra SK Swedo SE. Children with obsessive-compulsive disorder: are they just “little adults”? *J Clin Invest.* (2009) 119:737–46. 10.1172/jci37563 19339765 PMC2662563

[10] Zohar J Greenberg B Denys D. Obsessive-compulsive disorder. In: Schlaepfer TE Nemeroff CB editors. *Neurobiology of Psychiatric Disorders, Handbook of Clinical Neurology 3rd Series.* Amsterdam: Elsevier Science Publishers B.V (2012). p. 375–90. 10.1016/B978-0-444-52002-9.00021-8 22608632

[11] Hanna GL Piacentini J Cantwell DP Fischer DJ Himle JA Van Etten M. Obsessive-compulsive disorder with and without tics in a clinical sample of children and adolescents. *Depress Anxiety.* (2002) 16:59–63. 10.1002/da.10058 12219336

[12] Storch EA Merlo LJ Larson MJ Geffken GR Lehmkuhl HD Jacob ML Impact of comorbidity on cognitive-behavioral therapy response in pediatric obsessive-compulsive disorder. *J Am Acad Child Adolesc Psychiatry.* (2008) 47:583–92. 10.1097/CHI.0b013e31816774b1 18356759

[13] Mataix-Cols D Isomura K Pérez-Vigil A Chang Z Rück C Larsson KJ Familial risks of Tourette syndrome and chronic tic disorders: a population-based cohort study. *JAMA Psychiatry.* (2015) 72:787–93. 10.1001/jamapsychiatry.2015.0627 26083307

[14] Pauls DL. An update on the genetics of Gilles de la Tourette syndrome. *J Psychosom Res.* (2003) 55:7–12. 10.1016/s0022-3999(02)00586-x 12842226

[15] Pauls DL Fernandez TV Mathews CA State MW Scharf JM. The inheritance of Tourette disorder: a review. *J Obsessive Compuls Relat Disord.* (2014) 3:380–5. 10.1016/j.jocrd.2014.06.003 25506544 PMC4260404

[16] Georgitsi M Willsey AJ Mathews CA State M Scharf JM Paschou P. The genetic etiology of Tourette syndrome: large-scale collaborative efforts on the precipice of discovery. *Front Neurosci.* (2016) 10:351. 10.3389/fnins.2016.00351 27536211 PMC4971013

[17] Scharf JM Yu D Mathews CA Neale BM Stewart SE Fagerness JA Genome-wide association study of Tourette’s syndrome. *Mol Psychiatry.* (2013) 18:721–8. 10.1038/mp.2012.69 22889924 PMC3605224

[18] Ercan-Sencicek AG Stillman AA Ghosh AK Bilguvar K O’Roak BJ Mason CE L-histidine decarboxylase and Tourette’s syndrome. *N Engl J Med.* (2010) 362:1901–8. 10.1056/NEJMoa0907006 20445167 PMC2894694

[19] Fernandez TV Sanders SJ Yurkiewicz IR Ercan-Sencicek AG Kim YS Fishman DO Rare copy number variants in Tourette syndrome disrupt genes in histaminergic pathways and overlap with autism. *Biol Psychiatry.* (2012) 71:392–402. 10.1016/j.biopsych.2011.09.034 22169095 PMC3282144

[20] Karagiannidis I Dehning S Sandor P Tarnok Z Rizzo R Wolanczyk T Support of the histaminergic hypothesis in Tourette syndrome: association of the histamine decarboxylase gene in a large sample of families. *J Med Genet.* (2013) 50:760–4. 10.1136/jmedgenet-2013-101637 23825391

[21] International Obsessive Compulsive Disorder Foundation Genetics Collaborative [IOCDF-GC], OCD Collaborative Genetics Association Studies [OCGAS]. Revealing the complex genetic architecture of obsessive-compulsive disorder using meta-analysis. *Mol Psychiatry.* (2018) 23:1181–8. 10.1038/mp.2017.154 28761083 PMC6660151

[22] Davis LK Yu D Keenan CL Gamazon ER Konkashbaev AI Derks EM Partitioning the heritability of Tourette syndrome and obsessive compulsive disorder reveals differences in genetic architecture. *PLoS Genet.* (2013) 9:e1003864. 10.1371/journal.pgen.1003864 24204291 PMC3812053

[23] Mahjani B Bey K Boberg J Burton C. Genetics of obsessive-compulsive disorder. *Psychol Med.* (2021) 51:2247–59. 10.1017/s0033291721001744 34030745 PMC8477226

[24] Leonard HL Lenane MC Swedo SE Rettew DC Gershon ES Rapoport JL. Tics and Tourette’s disorder: a 2- to 7-year follow-up of 54 obsessive-compulsive children. *Am J Psychiatry.* (1992) 149:1244–51. 10.1176/ajp.149.9.1244 1503140

[25] do Rosario-Campos MC Leckman JF Curi M Quatrano S Katsovitch L Miguel EC A family study of early-onset obsessive-compulsive disorder. *Am J Med Genet B Neuropsychiatr Genet.* (2005) 136b:92–7. 10.1002/ajmg.b.30149 15892140

[26] van Grootheest DS Cath DC Beekman AT Boomsma DI. Twin studies on obsessive-compulsive disorder: a review. *Twin Res Hum Genet.* (2005) 8:450–8. 10.1375/183242705774310060 16212834

[27] Yu D Mathews CA Scharf JM Neale BM Davis LK Gamazon ER Cross-disorder genome-wide analyses suggest a complex genetic relationship between Tourette’s syndrome and OCD. *Am J Psychiatry.* (2015) 172:82–93. 10.1176/appi.ajp.2014.13101306 25158072 PMC4282594

[28] Browne HA Gair SL Scharf JM Grice DE. Genetics of obsessive-compulsive disorder and related disorders. *Psychiatr Clin North Am.* (2014) 37:319–35. 10.1016/j.psc.2014.06.002 25150565 PMC4143777

[29] Coffey BJ Miguel EC Biederman J Baer L Rauch SL O’Sullivan RL Tourette’s disorder with and without obsessive-compulsive disorder in adults: are they different? *J Nerv Ment Dis.* (1998) 186:201–6. 10.1097/00005053-199804000-00001 9569887

[30] Schrag A Martino D Apter A Ball J Bartolini E Benaroya-Milshtein N European multicentre tics in children studies (EMTICS): protocol for two cohort studies to assess risk factors for tic onset and exacerbation in children and adolescents. *Eur Child Adolesc Psychiatry.* (2019) 28:91–109. 10.1007/s00787-018-1190-4 29982875 PMC6349795

[31] Mataix-Cols D Frans E Pérez-Vigil A Kuja-Halkola R Gromark C Isomura K A total-population multigenerational family clustering study of autoimmune diseases in obsessive-compulsive disorder and Tourette’s/chronic tic disorders. *Mol Psychiatry.* (2018) 23:1652–8. 10.1038/mp.2017.215 29133949 PMC5951741

[32] Attwells S Setiawan E Wilson AA Rusjan PM Mizrahi R Miler L Inflammation in the neurocircuitry of obsessive-compulsive disorder. *JAMA Psychiatry.* (2017) 74:833–40. 10.1001/jamapsychiatry.2017.1567 28636705 PMC5710556

[33] Orlovska S Vestergaard CH Bech BH Nordentoft M Vestergaard M Benros ME. Association of streptococcal throat infection with mental disorders: testing key aspects of the pandas hypothesis in a nationwide study. *JAMA Psychiatry.* (2017) 74:740–6. 10.1001/jamapsychiatry.2017.0995 28538981 PMC5710247

[34] Schrag AE Martino D Wang H Ambler G Benaroya-Milstein N Buttiglione M Lack of association of group a streptococcal infections and onset of tics: European multicenter tics in children study. *Neurology.* (2022) 98:e1175–83. 10.1212/wnl.0000000000013298 35110379

[35] Swedo SE Leonard HL Garvey M Mittleman B Allen AJ Perlmutter S Pediatric autoimmune neuropsychiatric disorders associated with streptococcal infections: clinical description of the first 50 cases. *Am J Psychiatry.* (1998) 155:264–71. 10.1176/ajp.155.2.264 9464208

[36] Marazziti D Mucci F Fontenelle LF. Immune system and obsessive-compulsive disorder. *Psychoneuroendocrinology.* (2018) 93:39–44. 10.1016/j.psyneuen.2018.04.013 29689421

[37] Williams KA Swedo SE. Post-infectious autoimmune disorders: Sydenham’s chorea, pandas and beyond. *Brain Res.* (2015) 1617:144–54. 10.1016/j.brainres.2014.09.071 25301689

[38] Fontenelle LF Albertella L Brierley ME Thompson EM Destrée L Chamberlain SR Correlates of obsessive-compulsive and related disorders symptom severity during the Covid-19 pandemic. *J Psychiatr Res.* (2021) 143:471–80. 10.1016/j.jpsychires.2021.03.046 33958180 PMC8548281

[39] Abdulkadir M Tischfield JA King RA Fernandez TV Brown LW Cheon KA Pre- and perinatal complications in relation to Tourette syndrome and co-occurring obsessive-compulsive disorder and attention-deficit/hyperactivity disorder. *J Psychiatr Res.* (2016) 82:126–35. 10.1016/j.jpsychires.2016.07.017 27494079 PMC5026935

[40] Geller DA Wieland N Carey K Vivas F Petty CR Johnson J Perinatal factors affecting expression of obsessive compulsive disorder in children and adolescents. *J Child Adolesc Psychopharmacol.* (2008) 18:373–9. 10.1089/cap.2007.0112 18759647 PMC2935829

[41] Brander G Rydell M Kuja-Halkola R Fernández de la Cruz L Lichtenstein P Serlachius E Association of perinatal risk factors with obsessive-compulsive disorder: a population-based birth cohort, sibling control study. *JAMA Psychiatry.* (2016) 73:1135–44. 10.1001/jamapsychiatry.2016.2095 27706475

[42] Rosso G Albert U Asinari GF Bogetto F Maina G. Stressful life events and obsessive-compulsive disorder: clinical features and symptom dimensions. *Psychiatry Res.* (2012) 197:259–64. 10.1016/j.psychres.2011.10.005 22370150

[43] Khosravani V Aardema F Samimi Ardestani SM Sharifi Bastan F. The impact of the coronavirus pandemic on specific symptom dimensions and severity in OCD: a comparison before and during Covid-19 in the context of stress responses. *J Obsessive Compuls Relat Disord.* (2021) 29:100626. 10.1016/j.jocrd.2021.100626 33520614 PMC7834974

[44] Lin H Williams KA Katsovich L Findley DB Grantz H Lombroso PJ Streptococcal upper respiratory tract infections and psychosocial stress predict future tic and obsessive-compulsive symptom severity in children and adolescents with Tourette syndrome and obsessive-compulsive disorder. *Biol Psychiatry.* (2010) 67:684–91. 10.1016/j.biopsych.2009.08.020 19833320 PMC2843763

[45] Lin H Katsovich L Ghebremichael M Findley DB Grantz H Lombroso PJ Psychosocial stress predicts future symptom severities in children and adolescents with Tourette syndrome and/or obsessive-compulsive disorder. *J Child Psychol Psychiatry.* (2007) 48:157–66. 10.1111/j.1469-7610.2006.01687.x 17300554 PMC3073143

[46] Bornstein RA Stefl ME Hammond L. A survey of Tourette syndrome patients and their families: the 1987 Ohio Tourette survey. *J Neuropsychiatry Clin Neurosci.* (1990) 2:275–81. 10.1176/jnp.2.3.275 2136086

[47] Findley DB Leckman JF Katsovich L Lin H Zhang H Grantz H Development of the Yale children’s global stress index (YCGSI) and its application in children and adolescents ith Tourette’s syndrome and obsessive-compulsive disorder. *J Am Acad Child Adolesc Psychiatry.* (2003) 42:450–7. 10.1097/01.Chi.0000046816.95464.Ef 12649632

[48] van den Heuvel OA van Wingen G Soriano-Mas C Alonso P Chamberlain SR Nakamae T Brain circuitry of compulsivity. *Eur Neuropsychopharmacol.* (2016) 26:810–27. 10.1016/j.euroneuro.2015.12.005 26711687

[49] Milad MR Rauch SL. Obsessive-compulsive disorder: beyond segregated cortico-striatal pathways. *Trends Cogn Sci.* (2012) 16:43–51. 10.1016/j.tics.2011.11.003 22138231 PMC4955838

[50] Stein DJ Costa DLC Lochner C Miguel EC Reddy YCJ Shavitt RG Obsessive-compulsive disorder. *Nat Rev Dis Primers.* (2019) 5:52. 10.1038/s41572-019-0102-3 31371720 PMC7370844

[51] Landau YE Steinberg T Richmand B Leckman JF Apter A. Involvement of immunologic and biochemical mechanisms in the pathogenesis of Tourette’s syndrome. *J Neural Transm (Vienna).* (2012) 119:621–6. 10.1007/s00702-011-0739-x 22139323 PMC3936959

[52] Figee M Luigjes J Smolders R Valencia-Alfonso CE van Wingen G de Kwaasteniet B Deep brain stimulation restores frontostriatal network activity in obsessive-compulsive disorder. *Nat Neurosci.* (2013) 16:386–7. 10.1038/nn.3344 23434914

[53] Menzies L Achard S Chamberlain SR Fineberg N Chen CH del Campo N Neurocognitive endophenotypes of obsessive-compulsive disorder. *Brain.* (2007) 130(Pt 12):3223–36. 10.1093/brain/awm205 17855376

[54] Worbe Y Gerardin E Hartmann A Valabrégue R Chupin M Tremblay L Distinct structural changes underpin clinical phenotypes in patients with Gilles de la Tourette syndrome. *Brain.* (2010) 133(Pt 12):3649–60. 10.1093/brain/awq293 20959309

[55] Worbe Y Marrakchi-Kacem L Lecomte S Valabregue R Poupon F Guevara P Altered structural connectivity of cortico-striato-pallido-thalamic networks in Gilles de la Tourette syndrome. *Brain.* (2015) 138(Pt 2):472–82. 10.1093/brain/awu311 25392196 PMC4306818

[56] Shephard E Stern ER van den Heuvel OA Costa DLC Batistuzzo MC Godoy PBG Toward a neurocircuit-based taxonomy to guide treatment of obsessive-compulsive disorder. *Mol Psychiatry.* (2021) 26:4583–604. 10.1038/s41380-020-01007-8 33414496 PMC8260628

[57] McGuire PK Bench CJ Frith CD Marks IM Frackowiak RS Dolan RJ. Functional anatomy of obsessive-compulsive phenomena. *Br J Psychiatry.* (1994) 164:459–68. 10.1192/bjp.164.4.459 8038933

[58] Wang Z Maia TV Marsh R Colibazzi T Gerber A Peterson BS. The neural circuits that generate tics in Tourette’s syndrome. *Am J Psychiatry.* (2011) 168:1326–37. 10.1176/appi.ajp.2011.09111692 21955933 PMC4246702

[59] Allen A King A Hollander E. Obsessive-compulsive spectrum disorders. *Dialogues Clin Neurosci.* (2003) 5:259–71. 10.31887/dcns.2003.5.3/aallen 22033547 PMC3181632

[60] Porta M Servello D Sassi M Brambilla A Defendi S Priori A Issues related to deep brain stimulation for treatment-refractory Tourette’s syndrome. *Eur Neurol.* (2009) 62:264–73. 10.1159/000235595 19690419

[61] Porta M Saleh C Zekaj E Zanaboni Dina C Bona AR Servello D. Why so many deep brain stimulation targets in Tourette’s syndrome? Toward a broadening of the definition of the syndrome. *J Neural Transm (Vienna).* (2016) 123:785–90. 10.1007/s00702-015-1494-1 26739445

[62] Johnson KA Fletcher PT Servello D Bona A Porta M Ostrem JL Image-based analysis and long-term clinical outcomes of deep brain stimulation for Tourette syndrome: a multisite study. *J Neurol Neurosurg Psychiatry.* (2019) 90:1078–90. 10.1136/jnnp-2019-320379 31129620 PMC6744301

[63] Wehmeyer L Schüller T Kiess J Heiden P Visser-Vandewalle V Baldermann JC Target-specific effects of deep brain stimulation for Tourette syndrome: a systematic review and meta-analysis. *Front Neurol.* (2021) 12:769275. 10.3389/fneur.2021.769275 34744993 PMC8563609

[64] Denys D Mantione M Figee M van den Munckhof P Koerselman F Westenberg H Deep brain stimulation of the nucleus accumbens for treatment-refractory obsessive-compulsive disorder. *Arch Gen Psychiatry.* (2010) 67:1061–8. 10.1001/archgenpsychiatry.2010.122 20921122

[65] Luyten L Hendrickx S Raymaekers S Gabriëls L Nuttin B. Electrical stimulation in the bed nucleus of the stria terminalis alleviates severe obsessive-compulsive disorder. *Mol Psychiatry.* (2016) 21:1272–80. 10.1038/mp.2015.124 26303665

[66] Denys D Graat I Mocking R de Koning P Vulink N Figee M Efficacy of deep brain stimulation of the ventral anterior limb of the internal capsule for refractory obsessive-compulsive disorder: a clinical cohort of 70 patients. *Am J Psychiatry.* (2020) 177:265–71. 10.1176/appi.ajp.2019.19060656 31906709

[67] Kuhn J Lenartz D Mai JK Huff W Lee SH Koulousakis A Deep brain stimulation of the nucleus accumbens and the internal capsule in therapeutically refractory Tourette-syndrome. *J Neurol.* (2007) 254:963–5. 10.1007/s00415-006-0404-8 17410328

[68] Greenberg BD Gabriels LA Malone DA Jr Rezai AR Friehs GM Okun MS Deep brain stimulation of the ventral internal capsule/ventral striatum for obsessive-compulsive disorder: worldwide experience. *Mol Psychiatry.* (2010) 15:64–79. 10.1038/mp.2008.55 18490925 PMC3790898

[69] Menchón JM Real E Alonso P Aparicio MA Segalas C Plans G A prospective international multi-center study on safety and efficacy of deep brain stimulation for resistant obsessive-compulsive disorder. *Mol Psychiatry.* (2021) 26:1234–47. 10.1038/s41380-019-0562-6 31664175 PMC7985042

[70] Lack CW. Obsessive-compulsive disorder: evidence-based treatments and future directions for research. *World J Psychiatry.* (2012) 2:86–90. 10.5498/wjp.v2.i6.86 24175173 PMC3782190

[71] Rapp AM Bergman RL Piacentini J McGuire JF. Evidence-based assessment of obsessive-compulsive disorder. *J Cent Nerv Syst Dis.* (2016) 8:13–29. 10.4137/jcnsd.S38359 27594793 PMC4994744

[72] Tyagi H Ogunbiyi O. Pharmacological management of Tourette’s syndrome comorbid with obsessive-compulsive disorder in adult patients. *BJPsych Open.* (2021) 7(Suppl. 1):S297–8. 10.1192/bjo.2021.788

[73] McDougle CJ Epperson CN Pelton GH Wasylink S Price LH. A double-blind, placebo-controlled study of risperidone addition in serotonin reuptake inhibitor-refractory obsessive-compulsive disorder. *Arch Gen Psychiatry.* (2000) 57:794–801. 10.1001/archpsyc.57.8.794 10920469

[74] Masi G Pfanner C Brovedani P. Antipsychotic augmentation of selective serotonin reuptake inhibitors in resistant tic-related obsessive-compulsive disorder in children and adolescents: a naturalistic comparative study. *J Psychiatr Res.* (2013) 47:1007–12. 10.1016/j.jpsychires.2013.04.003 23664673

[75] Osland ST Steeves TD Pringsheim T. Pharmacological treatment for attention deficit hyperactivity disorder (ADHD) in children with comorbid tic disorders. *Cochrane Database Syst Rev.* (2018) 6:Cd007990. 10.1002/14651858.CD007990.pub3 29944175 PMC6513283

[76] Pringsheim T Okun MS Müller-Vahl K Martino D Jankovic J Cavanna AE Practice guideline recommendations summary: treatment of tics in people with Tourette syndrome and chronic tic disorders. *Neurology.* (2019) 92:896–906. 10.1212/wnl.0000000000007466 31061208 PMC6537133

[77] Williams T Shafran R. Obsessive–compulsive disorder in young people. *BJPsych Adv.* (2015) 21:196–205. 10.1192/Apt.Bp.113.011759

[78] Piacentini J Woods DW Scahill L Wilhelm S Peterson AL Chang S Behavior therapy for children with Tourette disorder: a randomized controlled trial. *JAMA.* (2010) 303:1929–37. 10.1001/jama.2010.607 20483969 PMC2993317

[79] Franklin ME Sapyta J Freeman JB Khanna M Compton S Almirall D Cognitive behavior therapy augmentation of pharmacotherapy in pediatric obsessive-compulsive disorder: the pediatric OCD treatment study II (POTS II) randomized controlled trial. *JAMA.* (2011) 306:1224–32. 10.1001/jama.2011.1344 21934055 PMC3495326

[80] Selles RR Højgaard D Ivarsson T Thomsen PH McBride NM Storch EA Avoidance, insight, impairment recognition concordance, and cognitive-behavioral therapy outcomes in pediatric obsessive-compulsive disorder. *J Am Acad Child Adolesc Psychiatry.* (2020) 59:650–9.e2. 10.1016/j.jaac.2019.05.030 31228561 PMC7179819

[81] Conelea CA Walther MR Freeman JB Garcia AM Sapyta J Khanna M Tic-related obsessive-compulsive disorder (OCD): phenomenology and treatment outcome in the pediatric ocd treatment study II. *J Am Acad Child Adolesc Psychiatry.* (2014) 53:1308–16. 10.1016/j.jaac.2014.09.014 25457929 PMC4254546

[82] Storch EA Jones AM Lack CW Ale CM Sulkowski ML Lewin AB Rage attacks in pediatric obsessive-compulsive disorder: phenomenology and clinical correlates. *J Am Acad Child Adolesc Psychiatry.* (2012) 51:582–92. 10.1016/j.jaac.2012.02.016 22632618

[83] Guzick AG Geller DA Small BJ Murphy TK Wilhelm S Storch EA. Irritability in children and adolescents with OCD. *Behav Ther.* (2021) 52:883–96. 10.1016/j.beth.2020.11.001 34134828 PMC8217718

[84] Bloch MH Leckman JF. Clinical course of Tourette syndrome. *J Psychosom Res.* (2009) 67:497–501. 10.1016/j.jpsychores.2009.09.002 19913654 PMC3974606

[85] Caurín B Serrano M Fernández-Alvarez E Campistol J Pérez-Dueñas B. Environmental circumstances influencing tic expression in children. *Eur J Paediatr Neurol.* (2014) 18:157–62. 10.1016/j.ejpn.2013.10.002 24210363

[86] Abrantes AM Strong DR Cohn A Cameron AY Greenberg BD Mancebo MC Acute changes in obsessions and compulsions following moderate-intensity aerobic exercise among patients with obsessive-compulsive disorder. *J Anxiety Disord.* (2009) 23:923–7. 10.1016/j.janxdis.2009.06.008 19616916

[87] Kim DD Warburton DER Wu N Barr AM Honer WG Procyshyn RM. Effects of physical activity on the symptoms of Tourette syndrome: a systematic review. *Eur Psychiatry.* (2018) 48:13–9. 10.1016/j.eurpsy.2017.11.002 29331594

[88] Freedman DE Richter MA. A narrative review of exercise and obsessive-compulsive disorder. *Gen Hosp Psychiatry.* (2021) 71:1–10. 10.1016/j.genhosppsych.2021.03.014 33887525

[89] Kandola A Stubbs B. Exercise and anxiety. *Adv Exp Med Biol.* (2020) 1228:345–52. 10.1007/978-981-15-1792-1_23 32342469

[90] Frank E Benabou M Bentzley B Bianchi M Goldstein T Konopka G Influencing circadian and sleep-wake regulation for prevention and intervention in mood and anxiety disorders: what makes a good homeostat? *Ann N Y Acad Sci.* (2014) 1334:1–25. 10.1111/nyas.12600 25532787 PMC4350368

[91] Rodrigo L Álvarez N Fernández-Bustillo E Salas-Puig J Huerta M Hernández-Lahoz C. Efficacy of a gluten-free diet in the Gilles de la Tourette syndrome: a pilot study. *Nutrients.* (2018) 10:573. 10.3390/nu10050573 29735930 PMC5986453

[92] Kendler KS. An historical framework for psychiatric nosology. *Psychol Med.* (2009) 39:1935–41. 10.1017/s0033291709005753 19368761 PMC2783473

[93] WHO. *The ICD-10 Classification of Mental and Behavioural Disorders.* Genève: World Health Organization (1993).

[94] Dalgleish T Black M Johnston D Bevan A. Transdiagnostic approaches to mental health problems: current status and future directions. *J Consult Clin Psychol.* (2020) 88:179–95. 10.1037/ccp0000482 32068421 PMC7027356

[95] Browne HA Hansen SN Buxbaum JD Gair SL Nissen JB Nikolajsen KH Familial clustering of tic disorders and obsessive-compulsive disorder. *JAMA Psychiatry.* (2015) 72:359–66. 25692669 10.1001/jamapsychiatry.2014.2656

[96] Ruscio AM Stein DJ Chiu WT Kessler RC. The epidemiology of obsessive-compulsive disorder in the national comorbidity survey replication. *Mol Psychiatry.* (2010) 15:53–63. 18725912 10.1038/mp.2008.94 PMC2797569

[97] Flament M Whitaker A Rapoport J Davies M Berg C Kalikow K Obsessive compulsive disorder in adolescence: an epidemiological study. *J Am Acad Child Adolesc Psychiatry.* (1988) 27:764–71. 3264280 10.1097/00004583-198811000-00018

[98] Heyman I Fombonne E Simmons H Ford T Meltzer H Goodman R. Prevalence of obsessive–compulsive disorder in the British nationwide survey of child mental health. *Br J Psychiatry.* (2001) 179:324–9. 11581112 10.1192/bjp.179.4.324

